# Determination of tryptophan and its indole metabolites in follicular fluid of women with diminished ovarian reserve

**DOI:** 10.1038/s41598-023-44335-9

**Published:** 2023-10-10

**Authors:** Ahui Liu, Haofei Shen, Qiuyuan Li, Juanjuan He, Bin Wang, Wenjing Du, Guangning Li, Mingtong Zhang, Xuehong Zhang

**Affiliations:** 1 Lanzhou University, Lanzhou, Gansu People’s Republic of China; 2 The First School of Clinical Medicine, Lanzhou University, Lanzhou, People’s Republic of China; 3 The First Hospital of Lanzhou University, Chengguan District, No. 1 Dong Gang Xi Road, Lanzhou, 730000 Gansu People’s Republic of China; 4 Key Laboratory for Reproductive Medicine and Embryo of Gansu Province, Lanzhou, People’s Republic of China; 5 Gansu Inspection and Testing Technical Engineering Laboratory for Chinese Herbal and Tibetan Medicine, NMPA Key Laboratory for Quality Control of TCM, Gansu Institute for Drug Control, No.7 Yin’an Road, An Ning District, Lanzhou, 730070 Gansu People’s Republic of China; 6 SCIEX, Beijing, China

**Keywords:** Endocrine system and metabolic diseases, Endocrine reproductive disorders, Reproductive disorders

## Abstract

Tryptophan (TRP) and its indole metabolites exhibit numerous biological effects, especially their antioxidant properties. This study used untargeted metabolomics in conjunction with targeted metabolomics to investigate the differential expression of tryptophan and its indole metabolites in follicular fluid (FF) of diminished ovarian reserve (DOR) and normal ovarian reserve (NOR) populations. This study included patients with DOR (n = 50) and females with NOR (n = 35) who received in vitro fertilization and embryo transfer. Untargeted metabolomics suggests that diminished ovarian reserve affects the metabolic profile of FF, TRP and indole metabolites were significantly down-regulated in the DOR group. Targeted metabolomics quantification revealed that the levels of TRP, IPA and IAA in the FF of the DOR group were significantly lower than those of the NOR group (*P* < 0.01). The concentration of TRP in FF is positively correlated with the available embryo rate in NOR females. These results provide data support to explore the pathogenesis of DOR and to look for new biomarkers and ovarian protectors. Additionally, alterations in TRP and its indole metabolites in FF may indirectly reflect the interaction between intestinal flora and the follicular microenvironment.

## Introduction

Diminished ovarian reserve (DOR) is defined as a decrease in the number and/or quality of oocytes^1^, leading to ovarian insufficiency and severely affects female fertility. The etiology of DOR remains elusive and may be related to genetic factors, medical factors such as radiotherapy or surgery, psychological and environmental factors. In recent years, the relationship between intestinal flora and metabolic status has attracted significant attention. It has been shown that aging is accompanied by changes in the diversity of the gut microbiota and an increase in the abundance of subdominant species, leading to a disruption in the balance between the gut microbiota and host metabolism^2^. Previous studies have shown complex interactions between gut microbial metabolites and ovarian follicular dysfunction^3^. Additionally, altered gut microbial metabolites and their associations with diseases, such as chronic kidney disease and Alzheimer's disease have been reported^4,5^. However, studies on the relationship between the metabolites of the intestinal flora and ovarian reserve function are still poorly understood.

Indole and its derivatives are tryptophan-derived metabolites from intestinal flora, such as indole-3-lactic acid (ILA), indole-3-propionic acid (IPA), indole acetic acid (IAA), and they are involved in the regulation of multiple biological functions including inflammation, oxidative stress, and neurotransmission^6,7^. Recent studies have found that IPA may be a potential biomarker for the development of diseases such as type 2 diabetes^8^, chronic kidney disease^9^, non-alcoholic fatty liver disease^10^, and cardiovascular disease^11^. There is also evidence that obesity and depression are associated with decreased levels of indoles^12,13^. However, whether intestinal flora-derived indole metabolites of tryptophan (TRP) are associated with ovarian function remains largely unknown.

Indole and its metabolites are absorbed by the intestinal epithelium and diffuse into the blood, where they can enter the systemic circulation and have multiple target organs^14^. The concentration of IPA in human serum is 1–10 µM under physiological conditions^15^. Follicular fluid (FF) provides the microenvironment for oocytes growth and maturation^16^, and is therefore a window into the metabolites secreted by oocytes and follicular cells. Investigation of changes in the metabolites of the FF may reveal potential factors that affect oocyte development in DOR patients. However, few studies have examined the expression of TRP and indole metabolites in FF.

Therefore, in the present study, untargeted metabolomics combined with targeted metabolomics was performed on FF from women with normal and diminished ovarian reserve function with the aim of: (1) Characterizing the metabolic differences of FF between the two groups. (2) Ultra performance liquid chromatography-tandem mass spectrometer (UPLC-MS/MS) to detect and quantitate the levels of TRP and indole metabolites in FF. (3) Explore the relationship between levels of TRP and indole metabolites in FF and embryo quality. This study aimed to reveal the association between the diminished ovarian reserve function and the levels of indole metabolites, which are produced from tryptophan by intestinal flora. These results will provide new data for the study of the pathogenesis of DOR, and search for potential biomarkers and ovarian protectors for clinical DOR patients.

## Materials and methods

### Study populations and sample collection

Patients with diminished ovarian reserve (DOR) (n = 50) and females with normal ovarian reserve (NOR) (n = 35) who received in vitro fertilization (IVF) or intracytoplasmic sperm injection (ICSI) and embryo transfer were recruited from the reproductive center of the First Hospital of Lanzhou University between June 2022 and October 2022. The current study was approved by the ethics committee of the First Hospital of Lanzhou University and obtained written informed consent from all the patients (Approval no. LDYYSZLLKH2023-02). We also confirm that all methods were performed in accordance with relevant guidelines and regulations. The diagnostic criteria for patients with DOR meet^17^ (1) and any two of (2): (1) Age ≤ 35 years; (2) requires two of the following three conditions to be met: 1) antral follicular count (AFC) < 5–7 follicles; 2) anti-Mullerian hormone (AMH) < 0.5–1.1 ng/ml; (3) basic follicle-stimulating hormone (FSH): 10–20 IU/L. The NOR patients included were infertility mainly due to tubal factors, specific diagnostic details are as follows: (1) Age < 35 years; (2) AFC: 8 < AFC < 24; (3) AMH: 2.0 ng/ml < AMH ≤ 6.8 ng/ml; (4) basic FSH < 10 IU/L, basic luteinizing hormone (LH) < 10 IU/L. Participants with endometriosis, polycystic ovary syndrome, chromosome abnormality, pelvic tuberculosis, history of ovarian surgery, and other endocrine diseases such as thyroid dysfunction, hyperprolactinemia, diabetes, etc., were excluded. All patients underwent controlled ovarian stimulation using GnRH-agonist short/long protocol or progesterone primed ovarian stimulation (PPOS) protocol. We obtained the medical history and clinical characteristics of the patients from the electronic medical records. Sample collection on the day of oocyte retrieval, the first tube of follicular fluid was obtained without contamination by blood, and then centrifuged at 3000 *rpm* for 10 min. The supernatants were finally collected and stored in a refrigerator at − 80 °C.

### Untargeted metabolomics analyses

#### Sample pre-processing and spectrum collection

Untargeted metabolomics analyses were performed in follicular fluid samples. The samples were taken at 200 μL and then added to 800 μL of acetonitrile/methanol (1:4, v/v) and vortex-mixed. Ultrasonic extraction (40 kHz) for 30 min at 4 °C was performed for all samples, followed by placing at − 20 °C for 30 min to sediment the protein. Centrifuged at 13,000 *g* for 5 min at 4 °C, then the supernatants were collected for LC–MS/MS analyses. Quality control (QC) samples were prepared by pooling equal volumes of all the samples and were regularly placed in the measurement sequence during the instrumental analysis to ensure the reliability and stability of the entire analytical process.

LC–MS/MS analyses were performed using a UHPLC-Q Exactive HF-X system (Thermo Fisher Scientific, Inc.). The chromatography was performed on an HSS T3 column (2.1 mm × 100 mm, 1.8 μm) at 40 °C. Mobile phase A consisted of 5% acetonitrile, 95% water, and 0.1% formic acid; mobile phase B of 47.5% acetonitrile, 47.5% isopropyl alcohol, and 5% water containing 0.1% formic acid. Gradient elution program was: 0–3.5 min, 0–24.5% B; 3.5–5 min, 24.5–65% B; 5–5.5 min, 65–100% B; 5.5–7.4 min, 100–100% B; 7.4–7.6 min, 100–51.5% B; 7.6–7.8 min, 51.5–0% B; 7.8–9 min, 0% B; 9–10 min, 0% B. The flow rate was set at 0.4 mL/min and the sample injection volume was 2 μL. A Thermo UHPLC-Q Exactive HF-X mass spectrometer equipped with an electrospray ionization (ESI) source running in either positive or negative ion mode was used for the mass spectrometric analysis. The ion spray voltage was 3500 V in the positive ion mode and − 3500 V in the negative ion mode. The sheath gas flow rate was 50 arb, and the aux gas flow rate was 13 arb. The capillary temperature was 325 °C. For targeted MS/MS analysis, rolling collision energy of 20, 40, and 60 V was used. Data was acquired in Data Dependent Acquisition mode. The mass scanning range is 70–1050 m/z.

#### Data processing and differential metabolite analysis

After data acquisition was completed, the peaks of the raw data were detected and calibrated to obtain a data matrix of retention times, mass-to-charge ratios, and peak intensities. The data is then checked for errors and missing values are filled. The response intensities of the mass spectrometric peaks were normalized by summation, with subsequent variables with relative standard deviation (RSD) > 30% of the QC samples were removed, and data were log10-transformed before analysis. The Human Metabolome Database (HMDB) (http://www.hmdb.ca) and Majorbio Database were utilized to retrieve and identify detected metabolites. All these steps were performed on the Majorbio Cloud Platform (https://cloud.majorbio.com). Next, The ropls (Version 1.6.2) in the R package were used for partial least squares discriminant analysis (PLS-DA) and orthogonal PLS-DA (OPLS-DA) analysis of the identified metabolites between the groups. The variable importance in the projection (VIP) value obtained from the OPLS-DA model was used to identify the contribution of each variable. Metabolites with VIP > 1.0 and P value < 0.05 were considered as metabolites with significantly different, and the results were visualized through a volcano plot.

### Quantitative assessment of tryptophan and its indole metabolites by UPLC-MS/MS

#### Reagents

TRP, IPA, and IAA were all purchased from Beijing Bailingwei Technology Co., Ltd (Beijing, China). Methanol, acetonitrile, and formic acid were purchased from Merck Chemicals (Darmstadt, Germany), and all reagents used were of analytical or HPLC grade.

#### Preparation of standard solution and sample

An appropriate amount of TRP, IPA and IAA reference were precisely weighed, and methanol was added to prepare the standard mother liquor (1000 ng/mL), respectively, which were stored at 4 °C for standby. For detection, the standard mother liquor was diluted at 80% (v/v) methanol/water to final concentrations of 1, 2, 5, 10, 20, 50, 100 and 500 ng/ml for the standard curve working solutions.

The 100 μL of FF sample was placed into a 1.5 mL EP tube, and 400 μL of extract solution (50% methanol:50% acetonitrile) was added. The tube was then vortex-mixed for 5 min and then the solution was kept at − 20 °C for 20 min. Blank FF samples from women with normal ovarian reserve. To avoid the interference of endogenous TRP, IPA, and IAA, the blank FF was prepared with activated charcoal^18,19^. The mixture was then stirred with a magnetic stirrer. Next, all the samples were centrifuged at 12,000 r/min at 4 °C for 15 min, and 100 μl of supernatant was taken and filtered with 0.2-μm microporous membrane. The filtrate finally collected was transferred into sample vials for chromatography.

#### The conditions of chromatography and mass spectrometry

Chromatographic separations were performed on an Eclipse Plus C18 column (2.1 mm × 100 mm, 1.8 μm) at 40 °C. Gradient elution was carried out by mobile phase A (0.1% formic acid and 5 mmol/L ammonium formate) and mobile phase B (acetonitrile). The elution program was as follow: 0 ~ 1 min, 90% A; 1 ~ 4 min, 90% A ~ 1% A; 4 ~ 5 min, 1% A; 5 ~ 5.1 min, 1% A ~ 70% A; 5.1 ~ 6 min, 70% A. The flow rate was 0.4 mL/min, and the injection volume of samples was 5 μL.

Mass spectrometry (MS) conditions were electrospray positive ion mode (ESI +), and multiple reaction monitoring (MRM) mode (Q1 mass (m/z)/ Q3 mass (m/z). The spray voltage was 3500 V and the ion source temperature (TEM) was set at 550 °C; the ion source gas 1 (GS1) and ion source gas 2 (GS2) were set at 55 psi and 45 psi, respectively; the curtain gas (CUR) was 30 psi. The specific MS parameters of each analyte are summarized in Table 1.

**Table 1:** The optimal mass spectrometry parameters for detecting tryptophan and its metabolites.

Analyte	Chemical formula	Q1 mass (m/z)	Q3 mass (m/z)	DP (V)	CE (V)	RT (time)
TRP	C11H12N2O2	205.1	188.1	70	14	0.85
IPA	C11H11NO2	190	130	104	28	3.53
IAA	C10H9NO2	176	130	96	20	3.31

*TRP* tryptophan, *IPA* indole-3-propionic acid, *IAA* indole-3-acetic acid, *DP* declustering potential, *CE* collision energy, *RT* retention time.

### Statistical analyses

Statistical analyses were conducted using IBM SPSS Statistics (25.0). Continuous variables were presented as means ± standard deviation (SD) and were assessed by Student t-test. Categorical variables were presented as numbers with percentages and compared using the Chi-square test. *P* < 0.05 were considered significant. In the present study, the TRP, IPA, and IAA concentrations in the NOR group were grouped using the tertile method (bottom tertile = T1, middle tertile = T2, top tertile = T3). A generalized additive mixed model (GAMM) was employed to observe the associations between the concentrations of the three metabolites in FF and the available embryo rate.

### Ethical approval

All clinical data were approved by the ethics committee of human assisted reproductive technology of Lanzhou University First Affiliated Hospital (Approval no. LDYYSZLLKH2023-02).

## Results

### Participant characteristics

A total of 85 individuals that fulfilled the inclusion criteria were included in this study, with 50 subjects in the DOR group, and 35 subjects in the NOR group. The baseline clinical characteristics and ovulation outcomes of the patients are summarized in Table 2. There was no significant difference between the DOR and NOR groups in terms of BMI, duration of infertility, primary infertility rate, basal LH and E2 levels, and ICSI rate (*P* > 0.05). The average age and basal FSH were higher in the DOR group compared to the NOR group (*P* < 0.05). However, the AMH, AFC, the number of > 16 mm follicles on the day of HCG, retrieved oocytes, mature oocytes, available embryos, and high-quality embryos were significantly lower in the DOR group than those in the NOR group.

**Table 2:** Comparison of clinical characteristics between the DOR and NOR groups.

Characteristics	DOR (n = 50)	NOR (n = 35)	*P*
Age (year)	31.28 ± 2.63	29.83 ± 2.94	0.019
Body mass index (kg/m^2^)	21.45 ± 2.32	20.73 ± 1.81	0.127
AMH (ng/mL)	0.80 ± 0.39	3.01 ± 0.99	< 0.001
AFC	5.84 ± 3.42	13.06 ± 3.63	< 0.001
Duration of infertility (y)	2.56 ± 1.64	2.64 ± 2.01	0.835
Primary infertility rate, n (%)	31 (62)	27 (77.14)	0.140
Baseline hormones			
FSH (mIU/ml)	9.32 ± 4.19	6.40 ± 1.55	< 0.001
LH (mIU/ml)	5.64 ± 3.37	5.34 ± 1.84	0.598
E2 (pg /ml)	42.39 ± 35.95	41.41 ± 17.24	0.881
> 16 mm follicles on the day of HCG (n)	4.44 ± 2.77	11.66 ± 5.03	< 0.001
No. of retrieved oocytes (n)	6.00 ± 3.98	17.29 ± 6.70	< 0.001
No. of mature oocytes (n)	4.82 ± 3.48	14.74 ± 6.69	< 0.001
No. of available embryos (n)	2.78 ± 2.67	8.43 ± 4.54	< 0.001
No. of high-quality embryos (n)	1.14 ± 1.63	3.74 ± 3.05	< 0.001

*AMH* anti-Mullerian hormone, *AFC* antral follicular count, *FSH* follicle-stimulating hormone, *LH* luteinizing hormone, *E2* estradiol.

### Multivariate statistical analysis of metabolites

In this part of the study, untargeted metabolomics analysis was performed on 20 individuals in each of the DOR and NOR groups. The basic information of the 40 subjects is shown in Supplementary Table S1. This experiment in positive and negative ion modes detected 3121 features; a total of 137 positive metabolites and 60 negative metabolites were finally identified. The Pearson correlation coefficient was analyzed based on the peak area value for QC samples. The results showed that there was a good Pearson's correlation between the QC samples, indicating the testing process is stable and the data reliability is high (Fig. S1A,B). Furthermore, the six QC samples were clustered tightly in PCA score plots, indicating the instrument stability is good (Fig. S1C,D).

**Figure 1:**
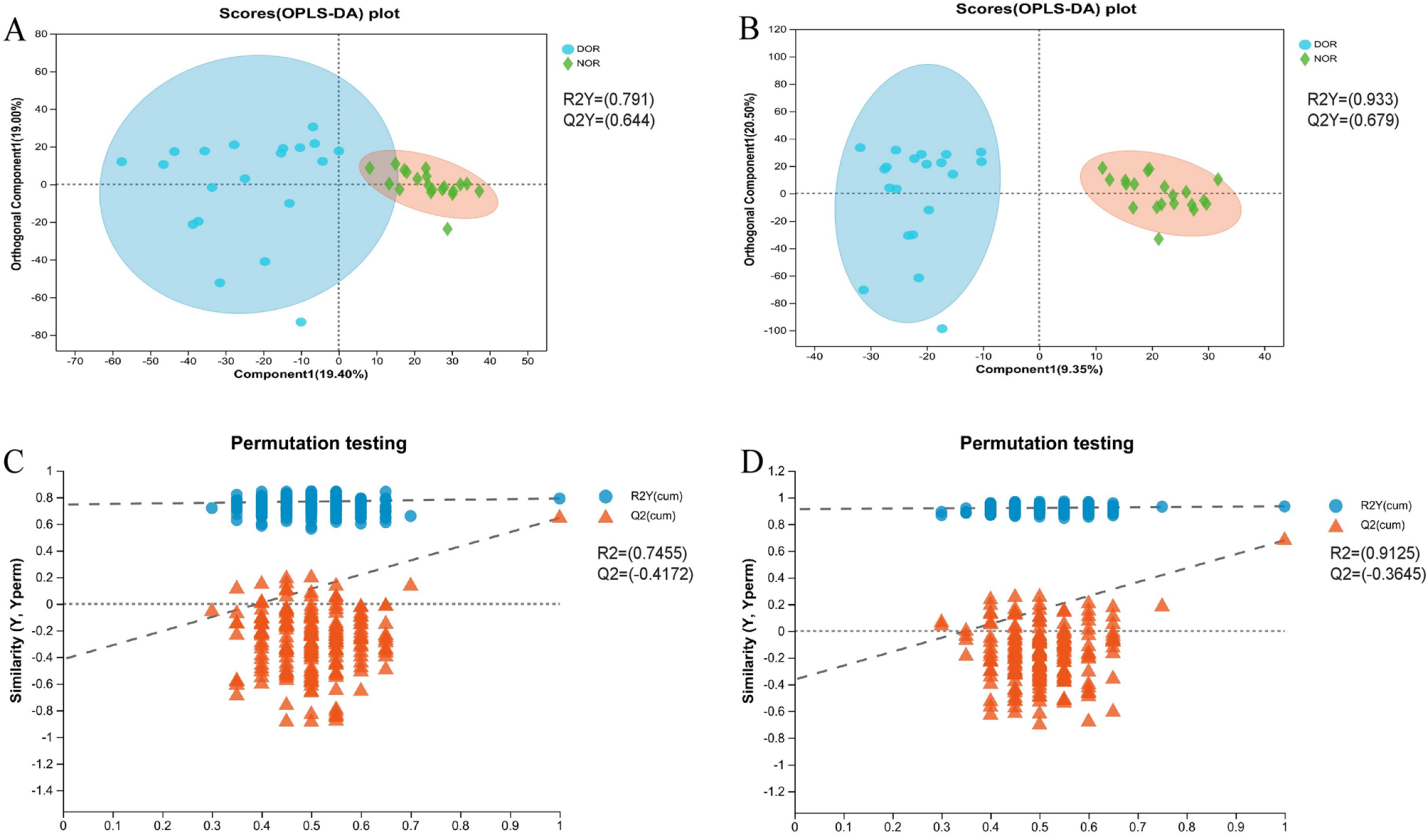
OPLS‑DA score plots and OPLS‑DA model permutation test derived from metabolomics profiles comparing the DOR and NOR groups. (**A**) OPLS-DA score plot in positive ion mode. (**B**) OPLS-DA score plot in negative ion mode. (**C**) OPLS-DA model permutation test in positive ion mode. (**D**) OPLS-DA model permutation test in negative ion mode.

To discriminate the degree of differences in metabolic profiles between the DOR and NOR groups, the PCA and OPLS-DA score plots of the two models were built. As presented in the OPLS-DA model (Fig. 1A,B), samples of the DOR group were clustered together, and all trended to the left, while the NOR clustered to the right. The values of R2Y and Q2Y are close to 1, indicating the effectiveness of the OPLS-DA model. The results of the permutation test further confirmed that the OPLS-DA model had good predictive ability and that there was no overfitting (positive mode: R2 = 0.7455, Q2 = − 0.4172; negative mode: R2 = 0.9125, Q2 = − 0.3645) (Fig. 1C,D). In addition, the PCA score plots also showed that the distribution of the two groups was different (Fig. S1C,D), but the two groups were not completely separated in the PCA model. These results also suggest that individual differences exist among the samples.

### Identification of differential metabolites and analysis of related items

**Figure 2:**
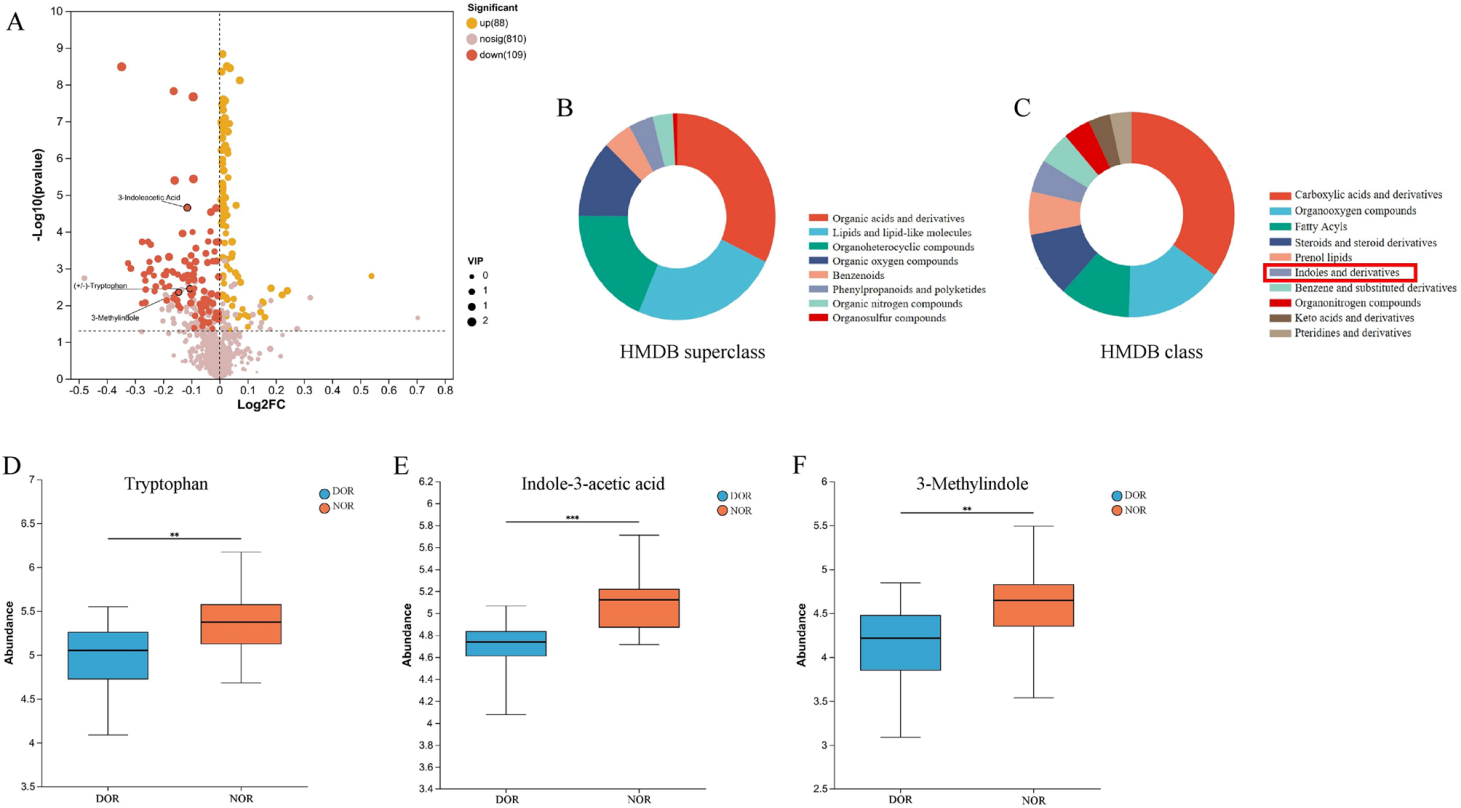
Untargeted metabolomics identifies metabolite changes in FF between the DOR and NOR groups. (**A**) The volcano plot to identify differential metabolomic profiles in FF. The three indole metabolites are labeled. (**B**) HMDB Superclass metabolites. (**C**) HMDB Class metabolites. (**D**–**F**) Details of the differences between groups [diminished ovarian reserve (DOR) = 20, normal ovarian reserve (NOR) = 20]. (**D**) Tryptophan. (**E**) Indole-3-acetic acid. (**F**) 3-Methylindole. Data are represented as boxplots. The line in the middle of the box represents the median, and the lines in the top and bottom represent the upper quartile and lower quartile, respectively. *P < 0.05, **P < 0.01, ***P < 0.001.

To further screen for differential metabolites, we performed univariate analysis. The criteria used in this project were a p-value of the Student's-test was less than 0.05, and the VIP value in the OPLS-DA model was greater than 1. Figure 2A shows a volcano plot of follicular fluid differential metabolite screening in the DOR and NOR groups. The light red area below the dotted line shows no statistically different metabolites (*P* > 0.05), and the upper dotted line shows statistically different metabolites (*P* < 0.05). A total of 88 up-regulated metabolites are shown in yellow, 109 down-regulated metabolites are shown in red. The dots size reflects the VIP value, and the larger the dot, the higher the VIP value. Furthermore, a heat map was used to visually represent the changes in metabolites between the DOR and NOR patients (Fig. S2).

We further categorized the identified metabolites by comparing the Human Metabolome Database (HMDB, http://www.hmdb.ca). Metabolites are categorized into 8 main items under the HMDB superclass and 10 items under the HMDB class (Fig. 2B,C). In particular, in the HMDB calss (Fig. 2C), Indole and derivatives are tryptophan-derived metabolites from the intestinal flora that have attracted our attention. Interestingly, as shown in Fig. 2A,D–F, all three indole metabolites including tryptophan, indole-3-acetic acid, and 3-Methylindole (3-MI) were significantly down-regulated in the DOR group.

### The concentrations of TRP and its indole metabolites in FF

**Figure 3:**
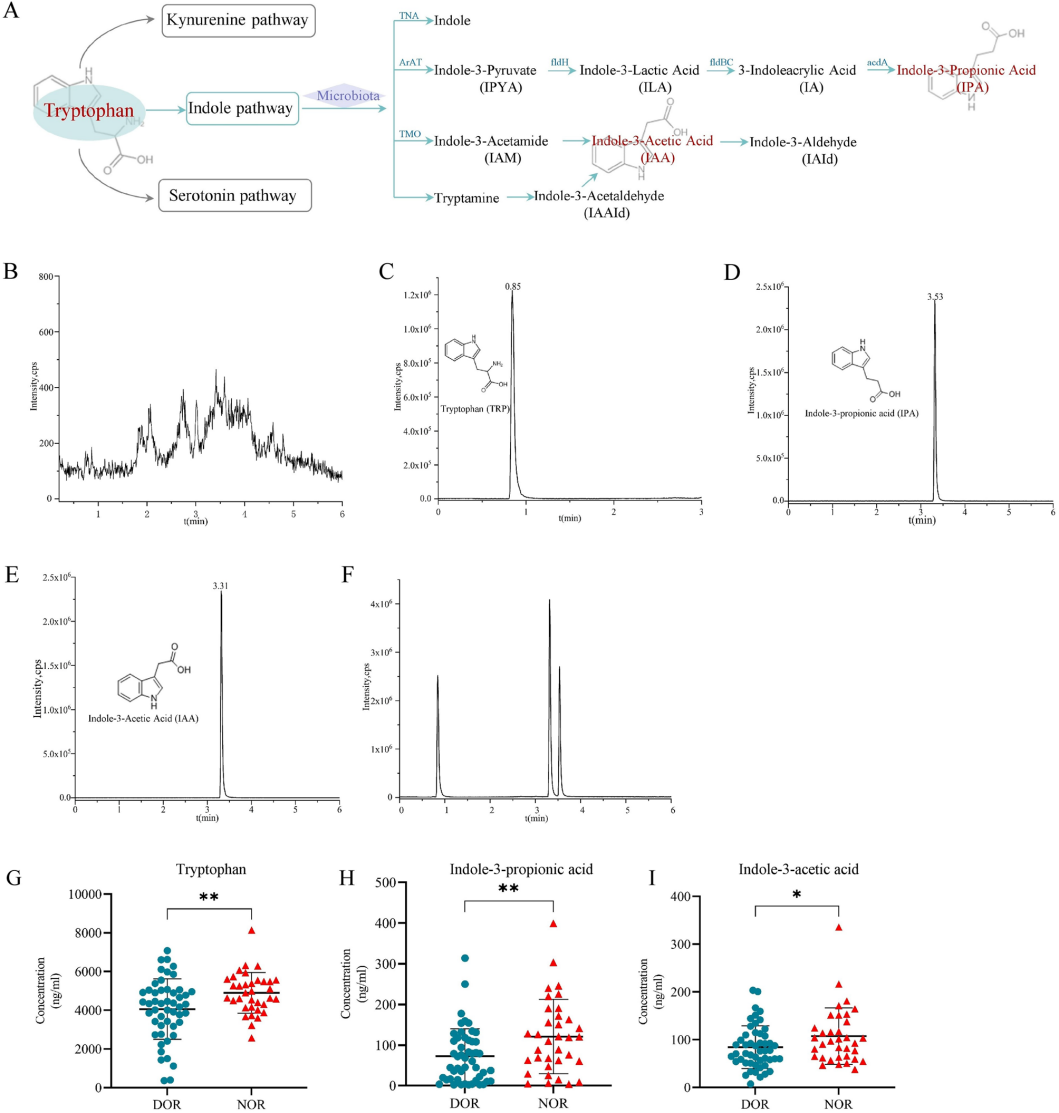
(**A**) Tryptophan metabolism pathways are shown. (**B**–**F**) The chromatograms of indole metabolites measured using UPLC-MS/MS. (**B**) The blank follicular fluid. (**C**) Tryptophan. (**D**) Indole-3-propionic acid. (**E**) Indole-3-acetic acid (**F**) Total ion flow. (**G**–**I**) Quantitative assessment of (**G**) Tryptophan, (**H**) Indole-3-propionic acid, and (**I**) Indole-3-acetic acid by UPLC-MS/MS. [diminished ovarian reserve (DOR) = 50, normal ovarian reserve (NOR) = 35].*P < 0.05, **P < 0.01, ***P < 0.001.

Based on the results of untargeted metabolomics, we focused on the indole metabolites of tryptophan and validated the relevant differential metabolites with targeted metabolomics. As shown in Fig. 3A**,** TRP is metabolized via three pathways: the kynurenine, the indole, and the serotonin pathway. In particular, the intestinal flora metabolism of tryptophan produced indole and many derivatives, including indole-3-propionic acid (IPA) and indole acetic acid (IAA), etc. In our experiments, the metabolites including TRP, IPA, and IAA were quantified by UPLC-MS/MS, and the chromatograms were shown in Fig. 3B–F. No significant peak was detected in the blank FF sample (Fig. 3B). The retention times for TRP, IPA, and IAA were 0.85 min, 3.53 min, and 3.31 min, respectively (Fig. 3C–E). The chromatograms of the three metabolites and the total ions have good peak shapes and were completely separated (Fig. 3F). The linear regression equation and coefficient of TRP, IPA, and IAA are as follows: y = 6448.711x + 4.585 × 10^4^ (r^2^ = 0.99743); y = 7204.64x − 1822.970 (r^2^ = 0.99951); y = 11,800.116x − 869.906 (r^2^ = 0.99903) (Table S2). The results showed that the compounds exhibited good linearity (r^2^ > 0.997) in a certain concentration range, and the precision and reproducibility of the assay were high.

Concentrations of the three metabolites in FF were recorded in a total of 85 individuals (50 were DOR, 35 were NOR). The results showed that the levels of TRP, IPA, and IAA in FF were significantly lower in the DOR group compared with the NOR group (Fig. 3G–I).

### Relationship between the three metabolites and available embryos

**Figure 4:**
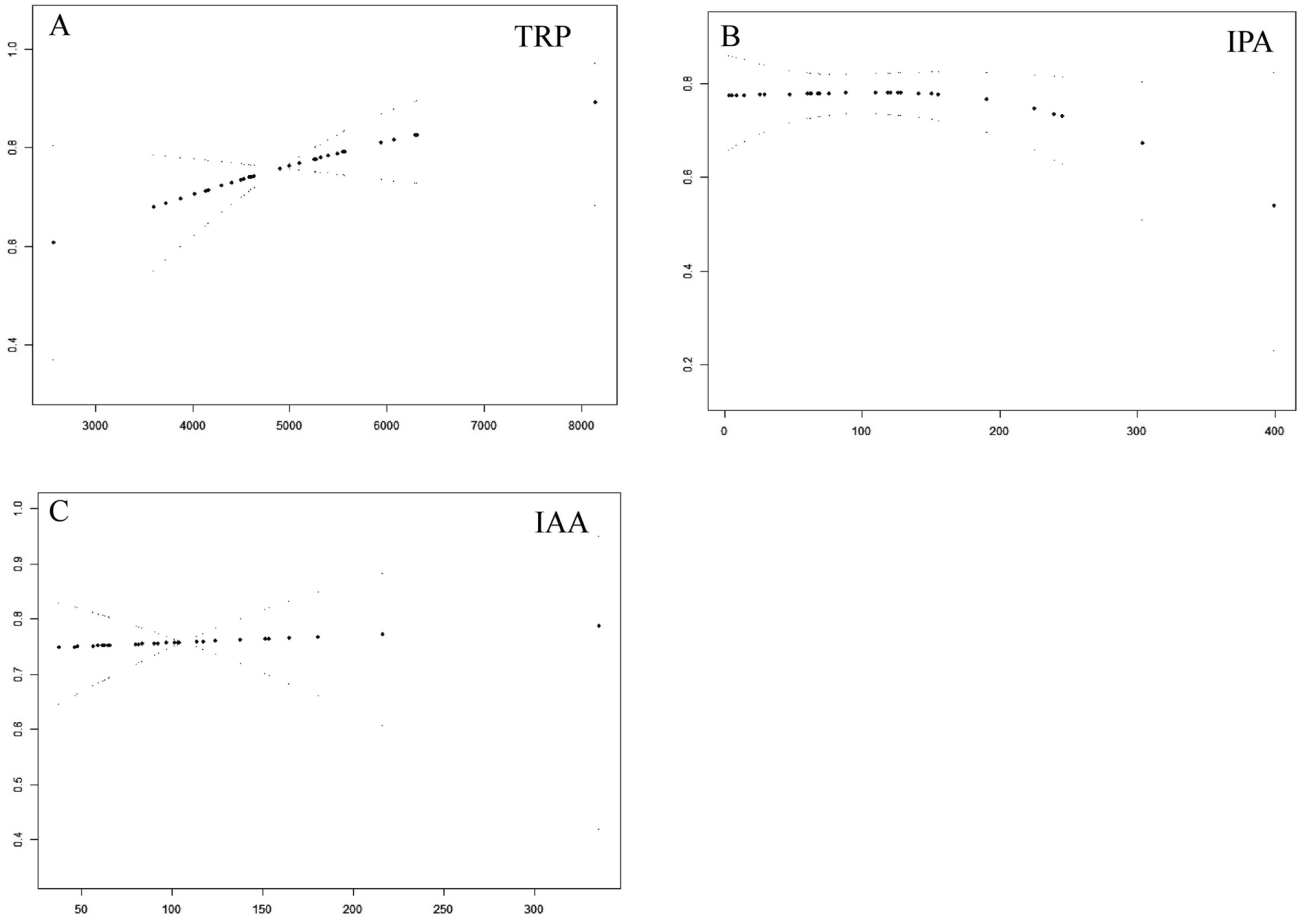
The GAMM model of the relationship between the concentrations of TRP (**A**), IPA (**B**), and IAA (**C**) in FF and the available embryo rate in the NOR group. The abscissa indicates the concentration of metabolites in the follicular fluid (ng/ml), and the ordinate indicates the available embryo rate in this IVF cycle.

Further GAMM curve analysis was used to investigate the relationship between the levels of the three metabolites including TRP, IPA, and IAA in FF and the available embryo rate in the NOR females. The results showed that the concentration of TRP was positively correlated with the available embryo rate (*P* < 0.05) (Table 3). There was no significant correlation between the concentration of either IPA or IAA and available embryos (*P* > 0.05) (Fig. 4A–C) (Table 3).

**Table 3:** The relationship between available embryos and the metabolites levels in the NOR group.

	T1	T2	T3	P value
TRP (ng/ml)	3600.40–4164.00	4584.00–5100.00	5552.00–6308.00	0.040
	65/99 (65.66%)	86/109 (78.90%)	87/110 (79.09%)	
IPA (ng/ml)	8.77–47.40	69.52–128.32	190.52–303.68	0.321
	71 (74.74%)	91 (79.13%)	76 (70.37%)	
IAA (ng/ml)	46.40–59.24	80.08–101.97	124.16–180.76	0.062
	60 (66.67%)	94 (81.03%)	84 (75.00%)

## Discussion

With the rapid development of biological analysis technology, metabolomics has become an important omics layer in multi-omics studies and has allowed us to explore the pathogenesis of DOR in greater depth. In recent years, studies have reported differences in FF metabolomics between DOR and NOR populations^20–22^. In the present study, we similarly found that diminished ovarian reserve function affects FF metabolic characteristics. HMDB was further used to classify the differential metabolites, which helped us to explore the biological significance of vital metabolites. Indoles and its derivatives in the HMDB class caught our special attention. Notably, TRP has three main metabolic pathways: the kynurenine pathway, the serotonin pathway, and the gut microbiota-derived indole pathway^23^. However, no differential metabolites of the other two pathways of TRP metabolism were detected in our experiment. Next, to further investigate the differences in indole metabolites in the FF between the two groups, we increased the sample size and applied a targeted metabolomics method, UPLC-MS/MS, to quantify TRP and the two major indole metabolites including IPA and IAA in FF and found that their concentrations were significantly lower in the DOR group, considering that they may be potential biological markers of DOR. Currently, UPLC-MS/MS has become the preferred method for the quantitative analysis of trace metabolites because of its high sensitivity, good selectivity, specificity, and fast analysis^24^.

TRP can be metabolized through these three pathways into various bioactive compounds such as serotonin, melatonin, NAD + and NAPD, which have important physiological functions for the body^25,26^. A growing number of studies also have found that TRP metabolites are associated with many diseases and dysfunctions such as Alzheimer's disease^5^, psychiatric disorders^27^, functional bowel disease^28^, and aging^29^. In recent years, some progress has been made in the study of TRP metabolism in the field of reproduction. As one of the main metabolites of TRP, serotonin regulates the secretion of steroid hormones and affects oocyte development and maturation^30^. In addition, many studies have reported on the antioxidant properties of TRP and its metabolites. Researchers have found that TRP and melatonin are important antioxidants in the human placenta, which can increase the expression of antioxidant enzymes in the placenta^31^. Remarkably, the application of melatonin in assisted reproduction achieved good results. Melatonin is localized in the FF and oocytes in mammals, protects cells from oxidative damage, and plays a beneficial role in oocyte maturation, fertilization, and embryonic development^32^. Tamura et al. reported that melatonin treatment in infertile patients can improve the quality of oocytes, thus increasing fertilization and pregnancy rates^33^. In this study, we found significantly lower levels of TRP and indole metabolites in the FF of DOR compared to NOR populations, and the concentration of TRP is positively correlated with the available embryo rate in NOR females. Our findings are consistent with a recent study on the metabolomics of FF in DOR patients, which found a similar decrease in TRP levels in the DOR group^21^. Taken together, it is reasonable to infer that TRP and its metabolites in FF have a protective effect on the ovary, the decrease in TRP and its degradation metabolites, especially in the FF of patients with DOR, may impair follicular quality, oocyte maturation and granulosa cell function, and may be detrimental to early embryos development. Notably, it is reported that more than 95% of TRP is degraded through the kynurenine pathway^26^. However, in this study, we did not detect differential metabolites associated with this pathway in the FF of the two groups. Does this suggest that TRP affects follicular development through the gut microbiota-derived indole pathway? More experiments are needed for further validation.

The most abundant metabolite of TRP in the intestine is indole, followed by IAA and IPA^34^, which are both decomposition products of the indole pathway described in Fig. 3A. IPA is a deamination product of TRP that has a heterocyclic aromatic ring with a similar chemical structure to melatonin^35^. In the last two decades, IPA has been shown to be an effective free radical scavenger^36^, which can protect neuronal cells from oxidative stress damage^37^. Recent studies have shown that IPA exerts beneficial effects in the immune^38^, gastrointestinal^34^, hepatic^39^, and cardiovascular^40^ systems by inhibiting inflammation, lipid peroxidation, and free radical formation. Rynkowska et al. found that IPA can effectively prevent membrane lipid oxidative damage caused by high iron concentrations in porcine skin^41^. Recently, two studies by Owumi SE's team demonstrated that exogenous administration of IPA attenuated toxicant-induced oxidative stress damage and apoptosis in testis and epididymis in male rats^42,43^. IAA, a phytohormone auxin, not only regulates plant senescence but its antioxidant and anti-inflammatory effects have also been revealed in cellular and animal experiments. It was reported that IAA could slow the progression of non-alcoholic fatty liver disease (NAFLD) in mice by inhibiting oxidative stress and inflammation^44^. An additional study by Kim et al. showed that IAA protected human dental pulp stem cells from H_2_O_2_-induced oxidative damage and this protection was achieved by activating the Nrf2-ARE pathway^45^. The targeted metabolomics results of this experiment showed that the levels of IPA and IAA were significantly lower in the FF of the DOR group than in the NOR group. Since indole metabolites have been poorly studied in ovarian function, we do not know whether IPA and IAA play a protective role in the ovary and how exactly they affect follicular development. As discussed earlier, the anti-oxidative stress and anti-inflammatory properties of IPA and IAA have been demonstrated in numerous experiments. We all know that oxidative stress damage is one of the important causes of DOR^46^. Oxidative stress may alter oocyte function, affect the FF microenvironment as well as the production of ovarian bioactive substances, and ultimately affect female reproduction. Therefore, we can make a reasonable assumption that IPA and IAA are absorbed by the intestinal epithelium and enter the systemic circulation, reaching the ovary and working with other substances to maintain cellular redox homeostasis, thus protecting the ovary from oxidative damage. However, further clinical and basic experiments are needed to verify the effects of indole metabolites on ovarian function, such as detecting the levels of malondialdehyde and catalase to confirm whether there is increased oxidative stress in DOR females.

Although the functional relevance of these two indole metabolites including IPA and IAA to ovarian function is currently unclear, our work also raises an intriguing possibility that the differences in IPA and IAA concentrations in FF indirectly reflect changes in the generation or utilization of intestinal microbial metabolites between DOR and NOR populations, as both IPA and IAA are tryptophan-derived metabolites from intestinal flora. It has been found that several genera and species of intestinal bacteria are involved in the synthesis of specific indole substances. The main bacteria involved in the production of IPA include L. reuteri^47^ and Clostridium genus^48^, while the major IAA-producing bacteria are Bacteroides, Clostridium and Bifidobacterium genus^49^. Both previous animal experiments and clinical studies have demonstrated that abnormal ovarian function such as premature ovarian failure (POF) and polycystic ovarian syndrome (PCOS) can alter the composition of the intestinal flora compared to controls with normal ovarian function^50,51^. With this in mind, we hypothesized that ovarian senescence may lead to a decrease in the intestinal flora that degrades indole metabolites, which in turn reduces the levels of indole metabolites such as IPA, and IAA in the FF and thus diminishes the protective effect on the ovary. In addition, another study analyzed the metabolomic changes in FF in obese and normal-weight women undergoing in vitro fertilization and found significantly lower levels of IPA in both FF and serum in obese women^52^, and the authors also considered that obesity and inflammation may modulate the gut microflora, leading to lower IPA levels. Taken together, it is also reasonable to consider that the intestinal flora may play an important role in regulating the levels of metabolites in FF. More research is necessary to further evaluate these hypotheses, such as analyzing differential gut microflora in DOR and NOR populations and exploring whether the specific flora is associated with differential metabolites in FF.

Nevertheless, there are still several limitations in our study. First, when conducting untargeted metabolomics analysis, both groups had a sample size of 20, which is a small sample study. Further study with a larger sample size is required to obtain more comprehensive information on differential metabolites in FF. Second, we measured only two indole metabolites (IPA and IAA) in FF by UPLC-MS/MS and did not quantify other indole metabolites such as 3-MI, ILA, and IAId. Besides, in the follow-up study, the levels of the above metabolites in serum should be analyzed to further clarify the levels of tryptophan and indole in the systemic circulation of DOR and NOR females. Third, further experiments, such as cellular and animal experiments, are needed to verify whether IPA and IAA can improve ovarian reserve function and their specific mechanisms, which will also help in clinical intervention and treatment of DOR.

In conclusion, this study employed untargeted metabolomics in conjunction with targeted metabolomics to focus on exploring the differential expression of tryptophan and its indole metabolites in FF of DOR and NOR populations. For the first time, we have used UPLC-MS/MS to quantify the levels of TRP, IPA and IAA in FF. These findings suggest that TRP and its indole metabolites may have potential antioxidant capacity in FF and indirectly reflect the interaction between intestinal flora and follicular microenvironment. Our results also provide data support to explore the pathogenesis of DOR and to look for new biomarkers and ovarian protectors.

### Supplementary Information

Supplementary Figures.

Supplementary Tables.

## Data Availability

The data used to support the findings of this study are available from the corresponding author upon request.
