# Supplementary material for: Determination of tryptophan and its indole metabolites in follicular fluid of women with diminished ovarian reserve
Source: Sci Rep. 2023 Oct 10;13:17124. doi: 10.1038/s41598-023-44335-9 (PMC10564947; doi:10.1038/s41598-023-44335-9)
Supplement: Supplementary file 1 — Supplementary Figures. [file 41598_2023_44335_MOESM1_ESM.docx]

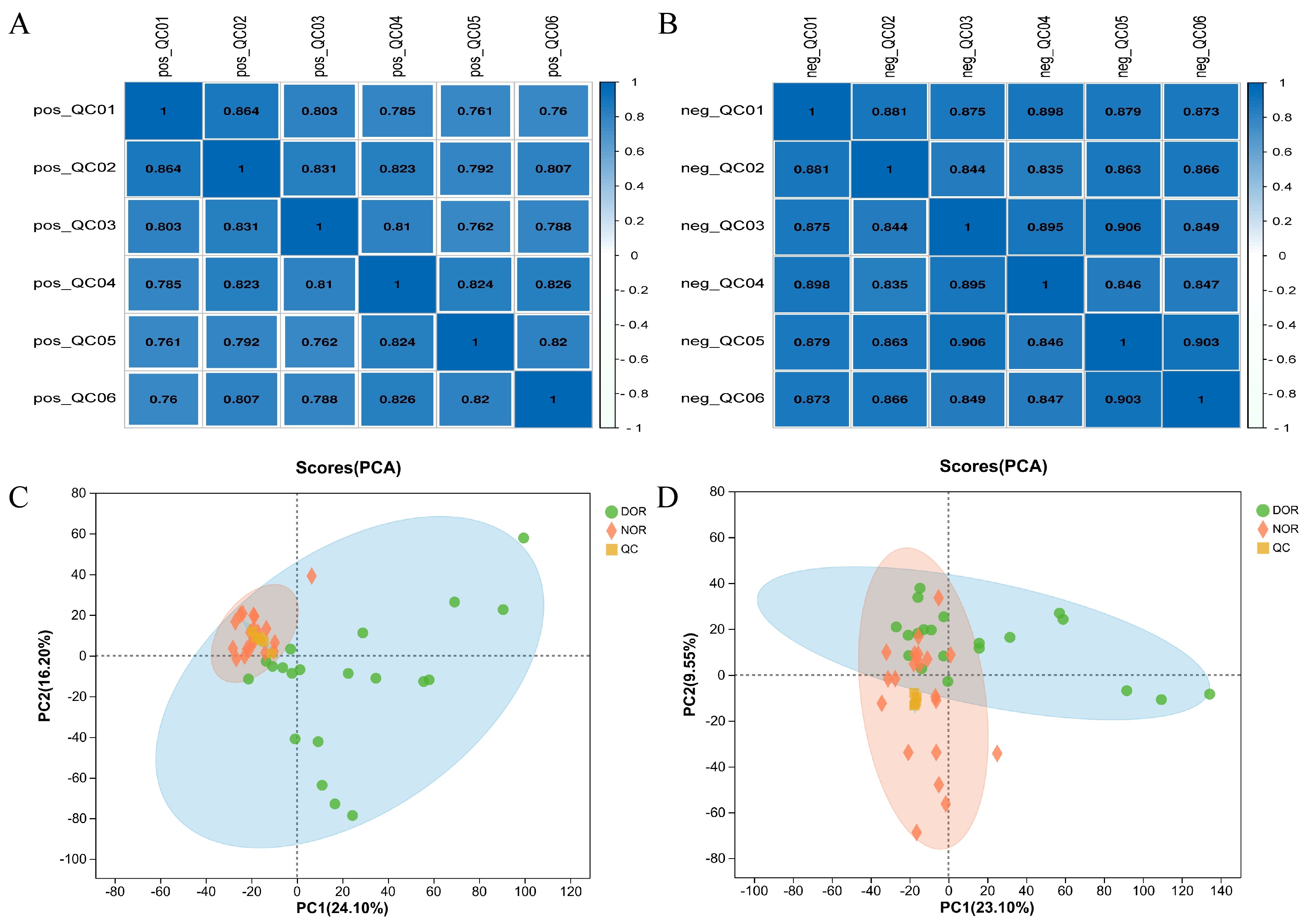


**Supplementary Fig 1 A-B:** Pearson correlation coefficient between the QC samples. **(A)** Positive ion mode. **(B)** Negative ion mode. **C-D:** PCA score plot with QC samples. **(C)** Positive ion mode. **(D)** Negative ion mode.


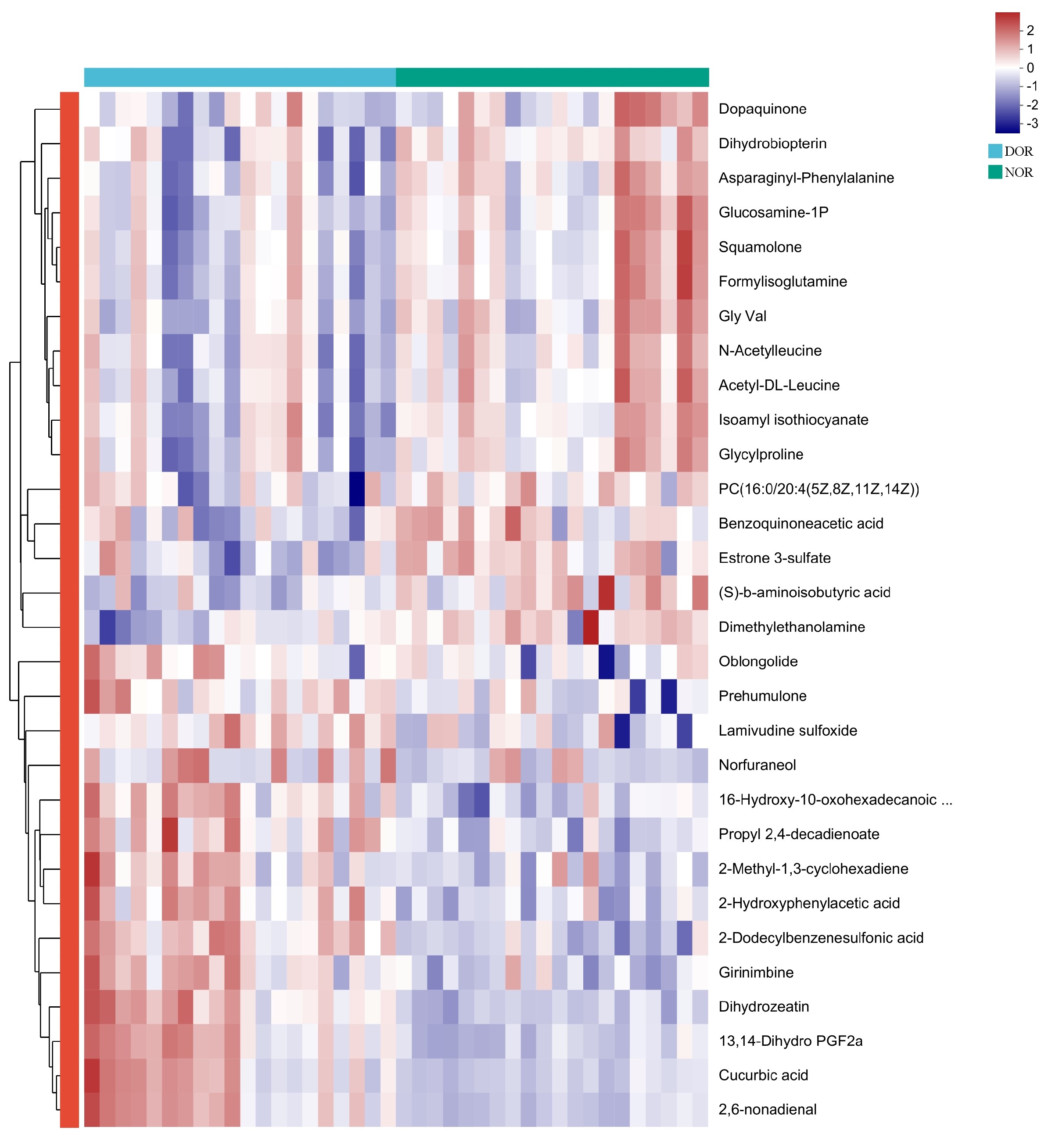


**Supplementary Fig 2** Heatmap based on 30 metabolites, unsupervised cluster analysis showed that differential metabolites could distinguish DOR patients from NOR females.
