# Supplementary material for: Determination of tryptophan and its indole metabolites in follicular fluid of women with diminished ovarian reserve
Source: Sci Rep. 2023 Oct 10;13:17124. doi: 10.1038/s41598-023-44335-9 (PMC10564947; doi:10.1038/s41598-023-44335-9)
Supplement: Supplementary file 2 — Supplementary Tables. [file 41598_2023_44335_MOESM2_ESM.docx]

**Table S1** Comparison of clinical characteristics of the two groups which performed untargeted metabolomics analysis.

| Characteristics | DOR (n=20) | NOR (n=20) | *P* |
| --- | --- | --- | --- |
| Age (year) | 32.90±1.45 | 31.60±1.88 | 0.019 |
| Body mass index (kg/m^2^) | 22.19±2.64 | 20.95±1.76 | 0.087 |
| AMH (ng/mL) | .84±0.34 | 3.12±1.08 | ＜0.001 |
| AFC | 5.03±2.83 | 12.35±3.42 | ＜0.001 |
| Duration of infertility (y) | 2.30±1.13 | 2.95±2.39 | 0.282 |
| Primary infertility rate, n (%) | 8 (40) | 14 (70) | 0.057 |
| Baseline hormones |  |  |  |
| FSH (mIU/ml) | 9.59±4.14 | 6.30±1.54 | 0.002 |
| LH (mIU/ml) | 5.72±1.94 | 4.60±1.51 | 0.048 |
| E2 (pg /ml) | 34.01±17.81 | 39.04±17.37 | 0.372 |
| >16 mm follicles on the day of HCG (n) | 4.05±2.72 | 10.95±5.69 | ＜0.001 |
| No. of retrieved oocytes (n) | 5.70±4.33 | 16.20±6.63 | ＜0.001 |
| No. of mature oocytes (n) | 4.40±3.84 | 13.55±6.41 | ＜0.001 |
| No. of available embryos (n) | 2.45±3.24 | 8.35±4.50 | ＜0.001 |
| No. of high-quality embryos (n) | 1.15±2.18 | 3.95±3.31 | 0.003 |

**Table S2** The linear regression equation and coefficient of TRP, IPA, and IAA

| Analyte | Linear regression equation | r^2^ | Linear range (ng /mL) |
| --- | --- | --- | --- |
| TRP | y=6448.711x+4.585×10^4^ | 0.99743 | 10-1000 |
| IPA | y=7204.64x-1822.970 | 0.99951 | 1-500 |
| IAA | y=11800.116x-869.906 | 0.99903 | 1-500 |
